# Caudal Additives Do Not Improve the Analgesia Afforded by Levobupivacaine After Hypospadias Repair

**DOI:** 10.5812/kowsar.22287523.2629

**Published:** 2012-01-01

**Authors:** Kay Davies, Graham Wilson, Thomas Engelhardt

**Affiliations:** 1 Department of Anesthesia, Royal Aberdeen Children’s Hospital, Aberdeen, UK

**Keywords:** Hypospadias, Analgesia, Pain, Child

## Abstract

**Background:**

Caudal analgesia is commonly employed to provide excellent intra- and postoperative analgesia for primary hypospadias repair in children. Several additives to local anesthetics are commonly employed to increase the block duration, although these have uncertain benefits.

**Objectives:**

This study investigated whether, in caudal analgesia with levobupivacaine 0.25%, the addition of S (+)-ketamine, clonidine, or both agents combined, would prolong postoperative analgesia in patients undergoing primary hypospadias repair.

**Patients and Methods:**

We conducted a retrospective chart analysis for all patients who underwent hypospadias repair with caudal analgesia over a consecutive 3-period at this institution. The study examined four patient groups, classified according to the analgesia used:

1. No additive, levobupivacaine alone
2. Levobupivacaine and S (+)-ketamine
3. Levobupivacaine and clonidine
4. Levobupivacaine, S (+)-ketamine, and clonidine

Primary outcome measures were as follows: time to the first postoperative request for analgesia, total first 24-hour postoperative analgesia, and time to hospital discharge.

**Results:**

The 87 patients included had a mean ± SD age of 21.4 ± 13.5 months and weight of 11.9 ± 2.4 kg. The median doses of levobupivacaine, S (+)-ketamine, and clonidine were 0.7 mg/kg (range, 0.4–1.3), 0.5 mg/kg (0.2–1.1), and 1.8 μg/kg (0.8–2.3), respectively. The addition of S(+)-ketamine, clonidine, or both did not increase the time to first oral analgesia request. Neither did it reduce the total first 24-hour postoperative analgesia requirements or alter hospital discharge time. However, the additive drugs in combination did increase postoperative sedation.

**Conclusions:**

The addition of S (+)-ketamine or clonidine to levobupivacaine 0.25% in caudal analgesia for hypospadias repair appears to be of no benefit. However, use of the additives in combination increased postoperative sedation.

## 1. Background

Single-shot caudal analgesia is routinely administered to provide optimal intra- and postoperative analgesia for primary hypospadias repair. Caudal analgesia provides excellent and predictable pain relief and is safe and easy to perform in children (1). A lumbosacral block can be obtained by using 0.5 mL/kg bupivacaine 0.25%, with an expected analgesic duration of 4 to 6 hours (1-3). Postoperative analgesia may be prolonged by a continuous infusion of local anesthetic or, potentially, by using additives. In the European Union, S (+)-ketamine and clonidine are now widely used as caudal additives, and evidence to support their use is available (4-12), particularly when used with racemic bupivacaine (4, 5, 7-10).

## 2. Objectives

This study tests the hypothesis that S (+)-ketamine or clonidine given separately or in combination in caudal analgesia with levobupivacaine 0.25% for primary hypospadias repair, prolongs the time to first analgesia request, reduces the total first 24-hour postoperative analgesia requirements, and shortens the time to hospital discharge.

## 3. Patients and Methods

Once approval from the regional ethics committee had been obtained, a retrospective analysis was undertaken of the records for three consecutive years of patients at the Royal Aberdeen Children’s Hospital who underwent primary hypospadias repair under caudal analgesia. The data were grouped into the following categories, according to the analgesia used:

1. No additive; levobupivacaine only
2. Levobupivacaine and S (+)-ketamine
3. Levobupivacaine and clonidine
4. Levobupivacaine, S (+)-ketamine, and clonidine

All caudal analgesia was administered after either intravenous or inhalational induction of general anesthesia, caudals were performed in the left lateral position under strict asepsis, using either a 22 G needle or the cannula technique. The local anesthetic used in the caudals was levobupivacaine 2.5 mg/mL (Chirocaine®). S(+)-ketamine and clonidine were administered as the preservative free-preparations Ketanest S® (5 mg/mL) and Catapres® (150 μg/mL), respectively.

Postoperative analgesia was administered on an “as required” basis by the nursing staff. Patients who scored 3 to 6 on the FLACC (face, legs, activity, crying, and consolability) pain scale were defined as having mild pain and were administered oral acetaminophen (20 mg/kg) and ibuprofen (5 mg/kg). Patients with moderate or severe pain received oral codeine phosphate (1 mg/kg) or oral morphine (0.3 mg/kg). Children undergoing hypospadias repair were routinely kept as inpatients for the first postoperative night.

Primary outcome measures were as follows: time to first postoperative analgesia request, total 24-hour postoperative analgesia requirement of acetaminophen and ibuprofen, and time to hospital discharge. Benzodiazepine premedication, intra-operative volatile agent, and the doses of levobupivacaine, S (+)-ketamine, and clonidine were recorded. In addition, any postoperative sedation, nausea and vomiting, or anti-emetic use was recorded, as well as the time to first oral intake. A child was considered to be sedated if the nurses had recorded his/her state as “drowsy” or “difficult to rouse.”

The Kruskal-Wallis test was used to compare median times to first analgesia request, median total doses of acetaminophen and ibuprofen required in the first postoperative 24 hours, median time to first oral intake, and median time to hospital discharge. The Chi-squared test was used to compare the incidence of postoperative sedation, postoperative nausea and vomiting, and use of anti-emetics among groups. P < 0.05 was considered significant.

## 4. Results

Over the 3-year period, 104 primary hypospadias repairs were performed. Records were found for 100 of these. Thirteen patients received intra- or postoperative morphine due to inadequate caudal analgesia or buccal mucosal grafts. The data on the remaining 87 patients, who received no other intra-operative analgesia, were analyzed. These patients had a mean ± SD age and weight of 21.4 ± 13.5 months and 11.9 ± 2.4 kg, respectively. The median and range of the doses of levobupivacaine, S(+)-ketamine, and clonidine for each group are shown in Table 1. Among the groups, there were no significant differences in the use of volatile agents or the duration of surgery. The same surgeon performed all the repairs.

**Table 1.** Drug Dose Categories for a Retrospective Evaluation of Analgesics for Hypospadias Repair

	LB ^a^, (n = 8)	LB and K ^a^, (n = 52)	LB and C a, (n = 15)	LB, K and C ^a^, (n = 12)
Levobupivacaine 0.25%, mL/kg, Median (range)	0.74 (0.47–1.28)	0.59 (0.44–1.02)	0.63 (0.41–0.94)	0.92 (0.73–1.03)
S (+)-ketamine, mg/kg, Median (range)	-	0.52 (0.15–1.06)	-	0.79 (0.5–1.03)
Clonidine, μg/kg, Median (range)	-	-	1.91 (1.4–2.34)	1.23 (0.73–1.03)

^a^ Abbreviations: C, clonidine; K, ketamine; LB, levobupivacaine

The addition of S (+)-ketamine, clonidine, or both to levobupivacaine 0.25% did not prolong the median time to administration of the first dose of postoperative analgesia (P = 0.52), nor did it reduce the median total first 24-hour postoperative analgesia requirement for acetaminophen (P = 0.15) or for ibuprofen (P = 0.14). Further, the median time to discharge was not reduced by the use of additives (P = 0.82) (Table 2). No child required postoperative analgesia other than acetaminophen or ibuprofen.

**Table 2.** Postoperative Analgesic Requirements for Hypospadias Repair, With and Without Additives

	LB ^a^, (n = 8)	LB and K ^a^, (n = 52)	LB and C ^a^, (n = 15)	LB, K, and C ^a^, (n = 12)
Time to first analgesia request, min, Median (range)	248 (150-365)	210 (75-900)	295 (65-4365)	253 (80-875)
Acetaminophen requirement in 24 hours, mg/kg, Median (range)	40.4 (22.5-85.6)	31.6 (14.1-81.8)	36.8 (13-67.4)	41.9 (19.1-56.1)
Ibuprofen requirement in 24 hours, mg/kg, Median (range)	4.3 (1.2-12.8)	8.4 (2-22.4)	11.4 (5.1-16)	9.2 (3-11.4)
Time to hospital discharge, min, Median (range)	1410 (220-1570)	1258 (235-7620)	1223 (400-4295)	1223 (265-11855)

^a^ Abbreviations:C, clonidine; K, ketamine; LB, levobupivacaine

The type of intra-operative volatile agent used had no influence on either the time to the first request for postoperative analgesia or the total dosage associated with the first postoperative 24 hours. Premedication with a benzodiazepine (midazolam 0.5 mg/kg) was only used for one patient in group 1 (levobupivacaine alone) and for seven patients in group 4 (levobupivacaine with both ketamine and clonidine). Although the use of benzodiazepine premedication prolonged the median time to the first postoperative analgesia request and reduced the total first 24-hour requirement for postoperative analgesia, the differences were not statistically significant (P = 0.64 and P = 0.69).

Although the combination of S (+)-ketamine and clonidine did produce postoperative sedation in significantly more patients (41.7%, P = 0.05) than the controls, there were no evident differences among the groups in terms of the time to first oral intake (P = 0.5), in the incidence of postoperative nausea and vomiting (P = 0.75), or in anti-emetic use (P = 0.09) (Table 3). 

**Table 3.** Postoperative Events in Hypospadias Repair Patients

	LB ^a^, (n = 8)	LB and K ^a^, (n = 52)	LB and C ^a^, (n = 15)	LB, K and C ^a^, (n = 12 ^b^)
Patients with postoperative sedation, No (%)	0	7 (13.5)	1 (13.3)	5 (41.7)
Median time to first oral intake, min, Median (range)	280 (55-500)	318 (75-555)	358 (165-550)	248 (125-1430)
Patients with postoperative nausea/vomiting, No (%)	1 (12.5)	2 (3.8)	1 (6.7)	1 (8.3)
Patients requiring anti-emetics, No (%)	1 (12.5)	0	1 (6.7)	0

^a^ Abbreviations: C, clonidine; K, ketamine; LB, levobupivacaine

^b^ Patients who received both K and C with the LB dose were significantly more sedated.

Overall, the incidence of postoperative nausea and vomiting was 5.7% and that of anti-emetic use was 2.3%. Pain scores and the quality of analgesia were not documented at the time of analgesia administration and thus could not be analyzed.

## 5. Discussion

This retrospective chart analysis demonstrates that, in children undergoing primary hypospadias repair, the addition of S (+)-ketamine and clonidine to the levobupivacaine 0.25% used to effect caudal analgesia does not prolong the time to the first request for analgesia or reduce the total 24-hour postoperative analgesia requirement for acetaminophen or ibuprofen. There was no significant difference in the median time to discharge among the groups, indicating no cost benefit for any of the techniques. These results suggest that S (+)-ketamine and clonidine, or the two in combination, when administered with levobupivacaine 0.25% during hypospadias repair, offer no additional analgesic benefit. We offer two possible explanations for these findings.

First, S (+)-ketamine and clonidine as caudal additives do not work well in combination with the S-enantiomer levobupivacaine and work better with the racemic bupivacaine preparation. Although considerable evidence supports the use of caudal S (+)-ketamine and clonidine with racemic bupivacaine (4, 5, 7-10), only limited evidence supports their use with levobupivacaine (13). That the S-enantiomer levobupivacaine is thought to have a superior safety profile, with a reduced local anesthetic toxicity, may explain the recent increase in its use in the UK (14). Surveys of UK pediatric anesthetists have shown a decrease in the use of racemic bupivacaine, from 94% in 2002 to 43.4% in 2009, with 41.7% of UK pediatric anesthesiologists now using levobupivacaine for caudal analgesia (15, 16). The efficacy of levobupivacaine in caudals has been proven by Frawley (17). For subumbilical surgery, Locatelli compared identical concentrations of caudally administered levobupivacaine, ropivacaine, and bupivacaine. Analgesic efficacy was similar among all three groups but bupivacaine resulted in a greater incidence of residual motor blockade and a longer analgesic block than the other two agents (18).

A second explanation for the results obtained in this study concerns the quality of postoperative analgesia provided by the caudally administered local anesthetic alone. Following hypospadias repair, levobupivacaine alone may be sufficient to render unnecessary further analgesia. Thus, the addition of S (+)-ketamine and clonidine, either alone or in combination, does not further enhance the already adequate analgesia. This idea is supported by a recent study of caudal analgesia for hypospadias repair that compared levobupivacaine 0.125% with levobupivacaine 0.375% (0.5 mL/kg body weight). Twelve of the 17 patients in this study were pain free at discharge on the morning following surgery when levobupivacaine 0.125% and no additional analgesic was used (19).

In the present study, neither S (+)-ketamine nor clonidine alone as caudal additive increased nausea, vomiting, anti-emetic use, or time to first oral intake, but when used in combination they did significantly increase postoperative sedation. Benzodiazepine premedication was given to just seven of these patients, yet 40% of the non-premedicated patients in this group exhibited postoperative sedation.

The main limitation of our study is its retrospective nature and the lack of consistently documented pain scores at the time of analgesia administration. Although nurses are under instructions to provide pain relief according to the severity of pain, its absence makes it impossible to comment on the quality of analgesia provided in each group. Assessment of pain in the age group studied is difficult, as it is frequently confounded in the postoperative period and confused with emergence delirium (20).

This study examined wide dose ranges for levobupivacaine, S(+)-ketamine, and clonidine, reflecting variations in the practice of individual anesthetists. Even so, the median doses of S (+)-ketamine and clonidine used were consistent with the recommended doses of caudally administered S (+)-ketamine (0.5–1 mg/kg) and clonidine (1–2 μg/kg).

In conclusion, the addition of S (+)-ketamine or clonidine to levobupivacaine used to induce caudal analgesia for primary hypospadias repair appears to offer no benefit. When used in combination, however, the two additives do significantly increase the percentage of patients with postoperative sedation.
